# Computational analysis to predict functional role of *hsa-miR-3065-3p* as an antiviral therapeutic agent for treatment of triple infections: HCV, HIV-1, and HBV

**DOI:** 10.3402/ljm.v7i0.19774

**Published:** 2012-12-31

**Authors:** Ambreen Khokhar, Samina Noorali, Muhammad Sheraz, Kuha Mahalingham, Donald G. Pace, Mohammad R. Khanani, Omar Bagasra

**Affiliations:** 1 Department of Pathology, Dow University of Health Sciences, Karachi, Pakistan; 2 South Carolina Center for Biotechnology, Claflin University, Orangeburg, SC, USA; 3 Department of English and Foreign Languages, Claflin University, Orangeburg, SC, USA

**Keywords:** HBV, HCV, HIV-1, homology, identity, inhibition, microRNA-based therapy, RNAi, triple infection

## Abstract

**Background:**

Triple infection (TI) with HIV-1, HCV, and HBV (TI) is highly prevalent in intravenous drug users (IDUs). These TI patients have a faster progression to AIDS, and even after antiretroviral therapy (ART) the prognosis of their disease is poor. The use of microRNA (miRNA) to silence genes holds potential applications for anti-HCV therapy.

**Methods:**

We analyzed the role of human miRNAs (*hsa-miRs*) in TI by computational analyses for HCV, HIV-1, and HBV showing identity to these three viral genomes.

**Results:**

We identified one unique miRNA, *hsa-miR-3065-3p*, that shares significant mutual identity to these three viral genomes (∼61–83%). In addition, *hsa-miR-99*, *hsa-miR-548*, and *hsa-miR-122* also showed mutual identity with these three viral genomes, albeit at a lower degree (∼52–88%).

**Conclusion:**

Here, we present evidence using essential components of bioinformatics tools, and hypothesize that utility of *hsa-miR-3065-3p* and perhaps miR-548 would be potential antiviral therapeutic agents in the treatment of TI patients because it shows near perfect alignment in the seed region for all three viruses. We also make an argument that current proposed therapy with *hsa-miR-122* may not be the optimal choice for HCV patients since it lacks essential gene alignment and may be harmful for the patients.

MicroRNAs (miRNAs) have emerged as key molecules in the regulation of a multitude of biological processes, from plants to animals, alongside approximately 20,000–30,000 coding genes (1–3). The regulation of miRNA expression is tightly controlled, and often the same rules and regulations that govern coding gene expression apply to miRNAs. Similar to coding genes, the alteration of levels of the temporal and differential expressions of each of the miRNA clearly affects the proper development and function of the tissue where it is expressed (4–6). MiRNAs, small 19–23 base pair (bp) double-stranded RNA molecules that are endogenously produced, are known to regulate at least one-third of all human genes (1, 4–9). They are also known to serve as a physiological and intracellular response to detect small double-stranded RNA (dsRNA), leading to the silencing or down-regulation in a sequence-specific manner of gene expression (3, 7). These RNA-based small molecules provide molecular immune defenses when the body is faced with challenges from transgene, RNA, or DNA viruses, as well as from transposons and aberrant miRNAs (3, 7–9). Both exogenous hosts in small interfering RNAs (siRNAs) and endogenously expressed miRNA molecules trigger RNA interference (RNAi) in eukaryotic cells (1–3). Until now (March 2012), researchers have identified 1,527 different human miRNAs (*hsa-miRs*) (Sanger Database version 18), and it has come to be regularly accepted that cellular gene regulation is significantly impacted by cellular miRNAs. Continually mounting evidence suggests that miRNAs control both RNA and DNA viruses by controlling their replications (9, 10). Evidence from our own laboratory has led us to believe that miRNAs can target the specific genetic material of invading viruses and serve as molecular immune systems (3, 8, 9). Triple infection (TI) with Human Immunodeficiency Virus Type-1 (HIV-1)/Hepatitis C Virus (HCV)/Hepatitis B Virus (HBV) are relatively less common among HIV-1 infected individuals, and these ‘triple’ infected individuals progress to AIDS relatively more quickly than mono or dual coinfected patients (e.g. HIV-1/HBV or HIV-1/HCV) (11–14). In our search for one or more *hsa-miRs* that may share mutual identity to all three viruses, and that can be utilized as a potential silencing agent for all three viruses in TI patients, we carried out alignments with the full-length genomes of HCV/HIV-1/HBV against the known *hsa-miRs* in the Sanger database. The well-characterized *hsa-mir-122* is also discussed here in the light of the new findings, including its role in HCV therapy (12, 13). Currently, *hsa-miR-122* is the major therapeutic miRNA against HCV, but it does not target the other two viruses. We present evidence that *hsa-miR-3065-3p* shares significant mutual identity with all three viruses, and that it may have a potential therapeutic application in TI patients, as well as in HCV mono-infected individuals (12–16).

## Materials & methods

### Mature miRNA database and gene alignment

We utilized alignment tools to discover miRNAs that share homologies to all three viral genomes. Finding miRNAs that share identities with all three viruses can potentially be a tool to quell the triple infection. At the time of our studies, 1,527 mature human miRNAs (hsa-miR) were listed in the Sanger database. By utilizing the human miRBase sequences database (http://www.mirbase.org, version 18.0), the hsa-miR sequences were aligned with each of the three viral genomes individually (i.e. HCV: Accession#NC_004102, HIV-1: Accession #NC_001802, and HBV: Accession #NC_003977). Before analysis, each U of hsa-miRs was converted to a T. The reference genome sequences of these three viruses were obtained from http://www.ncbi.nlm.nih.gov/. Following this, we utilized multiple alignment tools to search for miRNA that shared identities with all three viruses as described previously (17).

### Determination of microRNA alignment to viral sequences

To determine the suitability of each of the *hsa-miRs* as a potential therapeutic agent, we developed and refined the algorithm that incorporates the three critical elements that increases the suitability of a miRNA as a successful silencing agent. These include: 1) the length of the complementary pairing of human miRNAs. In this case, we downloaded the available miRNAs from miRbase data and aligned with HCV, HIV-1, and HBV genomes, as miRNA silencing requires a minimum of 19 bp (9, 17, 18); 2) a perfect or near-perfect alignment at miRNA seed sequences located at the 3’-untranslated region (3'UTR) base pairs 2–8 (9, 18) further signals a successful silencing match, and 3) a hairpin stem-loop secondary structure (4–6). Recently, the above information was detailed by us elsewhere (9, 18). The 80% degree level for the identity of the sequence of miRNAs with the three virus genome was considered as significant.

### Hairpin stem-loop secondary structure

**Fig. 1:**
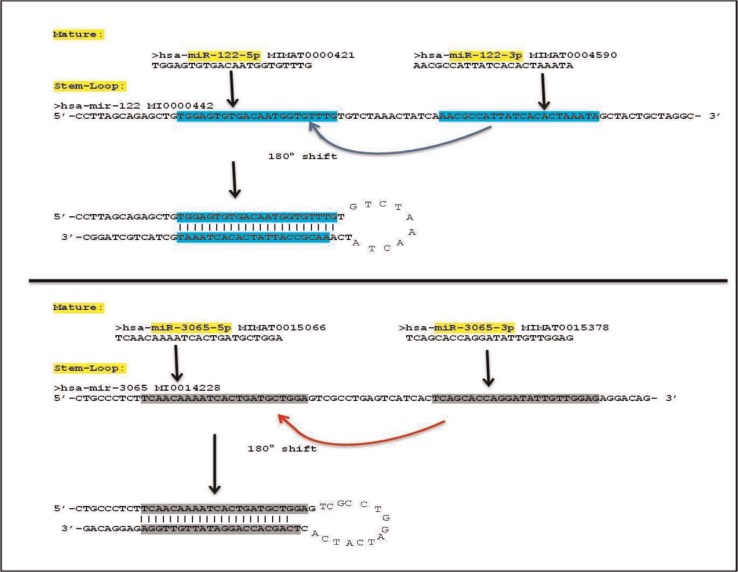
miRNAs are initially transcribed as several hundred nucleotide long primary or pri-miRNAs and are then processed to approximately 60-nucleotide (nt) hairpin pre-miRNAs in the nucleus by the double-stranded RNA (dsRNA)-specific ribonuclease (Drosha). The ribonuclease Drosha requires a dedicated dsRNA-binding protein to convert long, nuclear pri-miRNA transcripts into shorter pre-miRNA hairpin stem-loops. The pre-miRNAs are exported to cytoplasm where they are further excised by a RNA-induced silencing complex (RISC) enzyme. These miRNAs are further processed into numerous specific 19 to 23 nt miRNAs, with the ability to target various endogenous and exogenous genes (8).

The ‘mature’ and ‘stem-loop’ sequences for *hsa-miR-122* and hsa-miR-3065 were obtained from the ‘miRBase’ database (http://www.mirbase.org). The ‘mature’ sequences of the miRNAs are part of their ‘stem-loop’ sequences. The mature sequences, *in vivo*, result from excision of the middle (loop) region and the end of the ‘stem-loop’ sequences by DROSHA, an enzyme that allows the export of miRNAs from nucleoplasm to cytoplasm. The miRBase database provides both mature as well as stem-loop sequences for each miRNA and two mature sequences (5’ and 3’) for every stem-loop sequence of a miRNA. When both 5’ and 3’ mature sequences were aligned with stem-loop sequences for the same miRNAs, ∼100% alignment was obtained with certain regions of the stem-loop sequences. The 5’ and 3’ mature sequences comprise the portions between the middle and the ends on a stem-loop sequence as shown in Fig. 1. The 5’ and 3’ mature sequences are almost always complementary so that they bind together and the middle portion of the stem-loop sequence bulges out, which is further removed by DROSHA and presumably degraded (7–9).

## Results

### Human miRNA alignment

The target genes for the *hsa-miRNAs* were identified by retrieving the whole genome for HCV, HIV-1, and HBV individually from PubMed (http://www.ncbi.nlm.nih.gov/pubmed/). Each human miRNA (hsa-miR) found within the Sanger database (version 18.0) was determined to be identical to the Coding DNA Sequence (CDS) of the target gene by matching with 19–23 bp. The lengths of the 19–23 bp hsa-miRs were identical to those of the target genes of the three viruses, as identified by alignments, according to the full length CDS of the three viral genes using the specific alignment tools.

### *hsa-miR-3065* identity with HCV, HIV-1, and HBV

Here, we show via computational analysis that *hsa-miR-3065-3p* could be used as a therapeutic marker for treatment of triple infections (i.e. HCV, HIV-1, and HBV). The main focus of this study is *hsa-miR-3065-3p* and not *hsa-miR-122*, *hsa-miR-99*, or *has-miR-548*.

Through the computational analyses described in the *Methods* section in this article, we found that 42 *hsa-miRs* showed ∼80% identity to HCV (Supplemental Table 1), 41 miRNAs exhibited ∼80% identity to HIV-1 (Supplemental Table 2), and 37 miRNAs shared ∼80% identity to HBV (Supplemental Table 3). We also performed computational analysis for 1,527 *hsa-miRs* to assess their alignment with all six genotypes of HCV and found that *hsa-miR-3065-3p* was the only miRNA that aligned with HCV genotype 1, as well as with HIV-1 and HBV. It has also been reported that HCV genotype 1 is globally prevalent (19–21). As summarized in Table 1, we also show that three *hsa-miR*s (*miR-3065-3p*, *miR-99*, and *miR-548*) exhibited a significant mutual identity to genomic sequences of these three viruses. In addition, the homologies in the seed sequences at 3'UTR bps of *hsa-miR*-*3065-3p* were also relatively good but not perfect as they exhibited a non-homologous base pair match at the seed residue position #7 for HCV (Table 1). In addition, we also discovered non-homologous bases at position #6 for HIV-1 and position #7–8 for HBV. Of note, the most important sequences that should be identical are the first four bps of the seed sequence (5, 6, 22, 23) between the hsa-miRNA and the target sequence. Therefore, as shown in Table 1, in the case of *hsa-miR*-*3065-3p*, evidence has shown perfect homologies for all three viruses at the first four bps of the seed sequences (i.e. 2–5 bp). Interestingly, *hsa-miR-122* exhibited imperfect homologies in the seed sequences for all three viruses, even for HCV (23). The seed region is the most evolutionarily conserved region of miRNAs and is the region most complementary to target sites in 3'UTRs. In many cases, only a seed match is considered sufficient for gene silencing. Many target prediction algorithms are based around such a model, though increasing evidence demonstrates that targeting can also be mediated through sites other than the 3′-UTR and that seed region base pairing is not always required (5, 6, 22–24). Therefore, despite the importance of the seed region, the 3′-end of a miRNA also contributes to effective binding in roughly 2% of all preferentially conserved sites (5, 6, 9, 23). Furthermore, some validated miRNA target sites do not have a complete seed match but instead exhibit 11–12 continuous base pairs in the central region of the miRNA (25). However, an overall high identity of *hsa-miR-3065-3p* to all three viruses may still make this miRNA a strong candidate against all three viruses. Of note, it is significant that anti-HCV and anti-HIV-1 effects discovered *in silico* may be effective enough to offset the two bps non-homologous pairing for HBV.

**Table 1:** Human miRNA showing significant mutual identity with HCV, HIV-1, and HBV

No.	miRNA	Sequence alignment	Gene	Seed sequence homology	% Identity
1	hsa-miR-3065-3p	2418 CACCAGAACATTG-TGGA 2434 HCV ||||||·|·|||| |||| 5 CACCAGGATATTGTTGGA 22	HCV-GP1	15/18	83.3
	hsa-miR-3065-3p	8416 CAGCAGCA-GATAGGGTGGGAG 8436 HIV-1 |||||·|| ||||··||·|||| 2 CAGCACCAGGATATTGTTGGAG 23	HIV-NEF	17/22	77.3
	hsa-miR-3065-3p	2058 CAGCACTCAGGCAAGCTATTCTGTGTTGGGG 2088 HBV |||||| ||·|·||| ||||||·| 2 CAGCAC-----CAGGATAT----TGTTGGAG 23	HBV-GP4	19/31	61.3
2	hsa-miR-122*	6406 ACGGCATTATGCACACT 6422 HCV |||·|||||| |||||| 2 ACGCCATTAT-CACACT 17	HCV-GP1	15/17	88.2
	hsa-miR-122*	1818 AACGACCCCTCGTCACAATAAAGA 1841 HIV-1 |||| ||·|··|||||·||||·| 1 AACG--CCATTATCACACTAAATA 22	HIV-GAG-POL	18/34	52.9
	hsa-miR-122*	2306 AATGCCCCTATCTTATCAACACTTCCGGAAACTA 2339 HBV ||·|||··|||| ||||| ||·|| 1 AACGCCATTATC------ACACT------AAATA 22	HBV-GP1/HBVGP4	17/24	70.8
3	hsa-miR-99a	7105 CCTTCGATCCG--CTTGTG 7121 HCV ||·|·|||||| |||||| 4 CCGTAGATCCGATCTTGTG 22	HCV-GP1	15/19	78.9
	hsa-miR-99a	5381 AGCCAGTAGATCCTAGA-CTAGAG 5403 HIV-1 |·||·|||||||| || ||·|·| 1 AACCCGTAGATCC-—GATCTTGTG 22	HIV-1VPR-VPU	17/24	70.8
	hsa-miR-99b	630 CAAGAT---TCCTATGGGAGTG HBV ||||·| |·|||||||··|| 64 1 CAAGCTCGCTTCTATGGGTCTG 22	HBVGP1/HBV-GP2	15/22	68.2
4	hsa-miR-548a	2202 ACCATCAATTA------CACCA 2217 HCV |||| |||||| ||||| 5 ACCA-CAATTACTTTTGCACCA 25	HCV-GP1	15/22	68.2
	hsa-miR-548b	2763 AAGAACCTCCATTCCTTTGG 2782 HIV |||||||||··||·||||·| 2 AAGAACCTCAGTTGCTTTTG 21	HIV-POL	16/20	80.0
	hsa-miR-548s	301 TGGCCAAAATTCGCAGT 317 HBV |||||||||·| ||||| 2 TGGCCAAAACT-GCAGT 17	HBV-GP1/HBV-GP2	15/17	88.2

### *hsa-miR-122* identity with HCV, HIV-1, and HBV

We focus on HCV and miRNA because the first targeted miRNA therapy for HCV, known as miravirsen or SPC3649, is now being tested in a Phase 3 clinical trial of previously untreated chronic hepatitis C (CHC) patients by Santaris Pharma. In 2009, miR-122-based molecular therapy was the first to utilize miRNA in clinical trials involving humans; in 2012, this therapy entered Phase 3 of its clinical trial (21, 24). The *hsa-miR-122* was named miravirsen by California based Santaris Pharma, which initiated the trials in 2009.

### *hsa-miRs: −99 and −548* identity with HCV, HIV-1, and HBV

The other two miRNAs, *hsa-miR*-*99* and *hsa-miR-548*, did show, respectively, 79 and 68% identity with HCV, but their identity with the other two viruses (HIV-1 and HBV) was only significant with other branches of *hsa-miRs* (Table 1), which may not be important in regulating the viral replications of any of the three viruses. For instance, *hsa-miR-99a* showed 79% identity to HCV and 71% identity to HIV-1; however, 68% identity to HBV was observed in relation to *hsa*-*miR-99b*, a branch of *miR-99*.

Similarly, *hsa-miR-548* also showed significant identity with each of the three viruses: 68% with HCV, 80% with HIV-1, and 88% with HBV, and their importance in HBV or HIV-1 treatment as a therapeutic marker cannot be ruled out as the identity between *hsa-miR-548b* and *hsa-miR-548s* and viral target sites is 80% and above (Table 1). Furthermore, miR-548 showed perfect alignment at the first four bases of the seed sequences, making it a significant anti-TI therapeutic miRNA.

## Discussion

In our current investigation, we have carried out alignments of human *hsa-miRNAs* currently in the miR database with full-length genomes of the three viruses that share mutual identities. Our analyses identified that *hsa-miR-3065-3p* shares significant mutual identity with all three viruses. The well characterized *hsa-mir-122* is also discussed here in the light of the new findings, including its role in HCV therapy (12, 13, 21, 22, 24). Currently, *hsa-miR-122* is the major therapeutic miRNA against HCV but it does not target other two viruses. We present evidence that *hsa-miR-3065-3p* may have a potential therapeutic application in TI patients, as well as in HCV mono-infected individuals (12–16).

### HCV

HCV is an RNA virus and a member of the *Flaviviridae* family that infects the liver (26). HCV is a major cause of CHC infection and of hepatocellular carcinoma (HCC: 25). According to the World Health Organization, ∼200 million people are infected with HCV and between three and four million persons are newly infected each year, which leaves a global total of 170 million chronic carriers at risk for developing liver cirrhosis and/or HCC (26).

### Triple infection and *hsa-miR-3065* identity

All TI viruses share similar modes of transmission that include blood to blood contact, sexual contact with an infected person, or vertical (mother to child) transmission. Pakistan has an overall HIV-1 prevalence of 0.1%, but within certain high risk groups, such as IDUs and MSM (men who have sex with men); concentrated HIV-1 epidemics exist in certain parts of Pakistan–Afghanistan border areas and amongst the refugees where the prevalence of HIV is 2–3% (4–6, 27, 28). Limited data exist on the prevalence of HIV-1, HCV, and HBV in neighboring Afghanistan (27, 28). IDUs, MSM, commercial sex workers, migrant workers, and prisoners are recognized as subgroups at high risk for TI among the Afghan natives and among refugee populations (27). A major high risk behavior observed among infected participants in this study was the high drug abuse in TI patients – 28.4%. Out of these drug users, some individuals reported only injecting drugs (IDUs, 12.8%) while others admitted to both smoking/inhaling as well as injecting drugs (18.5%). A significant correlation was observed between drug use and HIV-1 infection in a recent study (27).

As shown in Table 1, *hsa-miR-3065-3p* exhibited significant identity with all three viruses. These identities were 83% for HCV, 77% for HIV-1, and 61% for HBV and provided a strong indication that the affinities between the inhibitory miRNA and the mRNAs of the homologous sequences in all three viral genomes may markedly silence all viruses. However, *hsa-miR-3065-3p* may indirectly be beneficial by silencing of HIV-1, which causes immunodeficiency and may be at least partially responsible for increasing the replications of HCV and HBV (29). Most importantly, for all three viruses, the seed sequence rules for efficient silencing described in the literature are perfectly satisfied, suggesting a strong silencing effect (5, 6, 9, 23).

Of note, miRNAs are double-stranded RNAs with single base overhangs at both ends; therefore, identities should have a similar impact on the mRNA degradation depending on the orientation of miRNA, binding as sense or antisense strands to the RNA-induced Silencing Complex (RISC) (30). The randomness of sense or antisense strands that enter the RISC to degrade a target mRNA requires analyses of identity of each miRNA (30).

### HCV and hsa-miR-122

An intense interest in the development of a miRNA-based therapy for HCV infection and *hsa-miR-122* has received considerable attention. The potential value of miRNA in antiviral immunity was first proposed in 1997 by Bagasra et al. (31, 31), but only very recently has the capacity of *hsa-miRNA* for both viral promotion and deterrence been demonstrated, mainly in the case of HCV infection (12, 13, 21–24, 32, 33). *Hsa-miR-122* is a liver-specific miRNA that positively regulates HCV RNA abundance and appears to be essential for the production of infectious HCV. A genetic approach has demonstrated that the ability of *hsa-miR-122* to enhance yields of the infectious virus is dependent upon two *hsa-miR-122*-binding sites near the 5’ end of the HCV genome, S1 and S2 (12, 21–24). Viral RNA with base substitutions in both S1 and S2 failed to produce infectious viruses in transfected liver cells, while the virus production was rescued to near-wild-type levels in cells supplemented with a complementary *hsa-miR-122* mutant. A comparison of mutants with substitutions in only one site revealed S1 to be dominant, but the S1 mutant did not produce high virus yields in liver cells supplemented with wild-type *hsa-miR-122*. According to Jangra *et al*. (12), translation of HCV RNA was reduced over 50% by mutations in either S1 or S2, and was partially rescued by the transfection of the complementary *hsa-miR-*122 mutant (12, 13). However, unlike the case for viral replication, both sites functioned equally in regulating translation. These investigators concluded that *hsa-miR*-*122* promotes replication by binding directly to both sites in the HCV genomic RNA and, at least in part, by stimulating internal ribosome entry site (IRES)-mediated translation (12). However, a comparison of the replication capacities of the double-binding-site mutant and an IRES mutant with a quantitatively equivalent defect in translation suggested that the decrement in translation associated with the loss of *hsa-miR-122* binding was insufficient to explain the profound defect in virus production by the double mutant. Hence, *hsa-miR-122* acts at an additional step in the virus life cycle by producing infectious HCV particles.

In a recent study (13), researchers investigated the effect of *hsa-miR-122* on the mevalonate pathway and showed that this miRNA does not affect HCV RNA abundance directly through mevalonate pathway in the animal liver. They determined that over-expression of *hsa-miR-122* enhanced viral RNA accumulation without affecting genes in the mevalonate pathway, such as the 3-hydroxy-3-methylglutaryl-coenzyme reductase (HMGCR) gene. However, inhibition of *hsa-miR-122* decreased both HCV RNA and HMGCR RNA abundance with little effect on the rates of HCV and HMGCR RNA synthesis, and loss of HCV RNA could not be restored by isoprenoid intermediate metabolites. Their findings suggest that *miR-122* modulates viral RNA abundance independently of its effect on isoprenoid metabolism. Of note, the mevalonate pathway is the main pathway that converts mevalonate to cholesterol and isoprenoid intermediates. These investigators (13) evaluated the physiological effects of *hsa-miR-122* sequestration and concluded that it decreased expression of transcripts encoding proteins of this pathway, such as HMGCR and Squalene epoxidase. Because these transcripts do not contain *hsa-miR-122-*binding sites, it is assumed that *hsa-miR-122* regulates the expression of HMGCR by the down-regulation of an inhibitor for these genes.

Currently, anti-HCV treatment based on miR-122 inhibition by antisense oligonucleotide anti-miR-122 (miravirsen or SPC3649) is the first miRNA-targeting treatment in human clinical trials to treat previously untreated CHC patients infected with HCV. Conversely, there is no miRNA-targeted therapy (21–24). Data from the Phase 2a study also showed that the mean change from baseline in HCV RNA (log10 IU/mL) at 10 weeks after initiation of therapy was −0.57, −2.16, and −2.73 in the 3, 5, and 7 mg/kg miravirsen dose groups, respectively, compared to the −0.01 level observed in the placebo group (21). Miravirsen was designed to recognize and interfere with *hsa-miR-122*, a liver-specific miRNA that HCV requires for replication. Studies to date have found that the drug appears safe, and that it reduces the HCV viral load in the liver and blood of chronically infected chimpanzees (21). On the contrary, for HIV-1 and HBV there is no miRNA-targeted therapy, and *hsa-miR-122* does show limited identities to HIV-1 and HBV (Table 1). Interestingly, *hsa-miR-122* is only 17 bp long, it has a non-homologous bp match at seed residue position #4 to HCV, and to be a potential candidate for gene-based therapy, the first four bps of the seed sequence should be identical (23, 24), which they are not (Table 1). Furthermore, a synergistic anti-TI effect of *hsa-miR-3065-3p* may inhibit the three viruses in a multi-target fashion, where downregulation of one virus may down-regulate the other two. Hence, through our computational analysis, we propose the utility of *hsa-miR-3065-3p* in TI patients as a potential therapeutic agent infected with HCV/HIV-1/HBV It is worth mentioning that *hsa-miR-122* has been the subject of intensive investigations around the globe, and it has been fairly well established that this miRNA appears to play a crucial role in upregulating intracellular HCV replication in hepatic cells (12, 13, 21, 22, 24). We also carried out genome/miRNA alignment to determine the degree of identity of *hsa-miR-122* and discovered that even though this miRNA does share a significant (∼88%) identity to HCV, it does not appear to follow the basic rules in relation to having the minimum 19 bp required for functional miRNA expression *in vivo* and *in vitro* (9, 21), since it has 17 miRNAs. In addition, the seed sequence rules for efficient silencing described in the literature are not entirely satisfied (5, 6, 9). Therefore, it lacks perfect identity at seed sequence bp #4 for HCV (located at 3'UTR 2–8). Moreover, it is 17 bp long and 15 nucleotides are homologous to HCV (Table 1), making it an unlikely candidate for therapeutic utility. Since this same miRNA may target many other mRNAs, unwanted side effects may occur (9). Furthermore, *hsa-miR-122* shares a much lesser identity to HIV-1 and HBV (53 and 71%, respectively), which potentially makes it less effective as a therapeutic treatment agent against TI patients (14–16). These data suggest the need for alternate miRNAs for targeting CHC subjects.

A study that investigated 185 seropositive patients’ sera for HCV and correlated *hsa-miR-122* expression and HCV load in liver samples found that hepatic *miR-122* expression was not correlated with the viral load (32). Therefore, *miR-122*, by itself, is not a critical molecular target for HCV therapy. *Hsa-miR-122* expression is inversely correlated with both functional and histopathological liver damage and is believed to stimulate HCV replication through interaction with two adjacent sites downstream of stem loop I (SLI) within the HCV 5'UTR. Recently, it has been demonstrated that although the approach of host-factor *hsa-miR-122* antagonism has potential for HCV therapy, and is being tested in clinical trials, the reduced antiviral effect by single mutation in S1 supports the reevaluation of this approach as monotherapy for future HCV treatment (32). Therefore, identification of host cellular RNA insertions in the HCV genome might facilitate understanding of virus–host interactions and can contribute to the identification of other host cellular interacting partners as future drug targets.

### *hsa-miRs:* −*99 and* −*548* identity with HCV, HIV-1, and HBV

The *hsa-miR*-*99* showed reasonable alignments of 79, 71, and 68% identities, respectively, with all three viruses but lacked significant identities at the seed sequences and was only significant with other branches of *hsa-miRs* (Table 1). This may not be important in regulating the viral replications of all three viruses *and* we did not consider it to be of any important therapeutic benefit.

On the contrary, *hsa-miR-548* showed 68, 80, and 88% identities with HCV, HIV-1, and HBV, respectively, and 100% identities at the seed sequence with the other two viruses (HIV-1 and HBV), which make it an important anti-TI miRNA, in particular, when it showed significant identities to HIV-1 and HBV (i.e. 80 and 88%, respectively). Since dual infection with HIV-1 and HBV are much more common than the TI, this miRNA can also be a valuable therapeutic agent in TI as well as in dual infected patients (22, 23). Li et al. (29) have shown that HIV-1 may accelerate HBV progression by lowering CD4 count, weakening HBV-specific immunity, increasing the development of HBV mutants, and causing immune activation. On the contrary, HBV may enhance HIV-1 replication by activating HIV long terminal repeat with X protein (HBX) and cause immune activation in synergy with HIV-1.

Liang et al. (34) have carried out a genome-wide analysis of *hsa-miR-548* and have shown that this miRNA belongs to a larger, poorly conserved primate-specific miRNA gene family. They detected 69 human mir-548 genes located in almost all human chromosomes whose widespread distribution pattern implicates the evolutionary origin from transposable elements. Higher level of nucleotide divergence was detected between these human miRNA genes, which mainly derived from divergence of multicopy pre-miRNAs and homologous miRNA genes. The location of mir-548 gene family members is detected in all human chromosomes except chromosomes 19 and *Y* and over 30% of the members are located in chromosomes 6, 8, and *X*. Furthermore, functionally miR-548 miRNAs are linked to various signaling pathways, including MARK, Wnt insulin, calcium, and p53 signaling pathways as well as to various cancers, including melanoma, colorectal, renal, small cell lung cancer, and glioma. However, no previous report has shown its involvement in quelling triple infections. Therefore, this is the first report where we are presenting potential use of miR-548 as a therapeutic agent in TI.

In this study, we present the theoretical evidence that by utilizing a strict thermodynamic algorithm, *hsa-miR-122* does not have the efficient HCV-promoting effect as has been suggested by numerous investigations (9, 12, 13, 21, 22, 24). It is also evident that the role of *hsa-miR-122* as a potential antiviral agent in HCV therapeutics still needs further investigation. We analyzed the identity of *hsa-miR-3065-3p* through computational analysis and came to the conclusion that it is perhaps a better miRNA than *hsa-miR-122* as a therapeutic agent for HCV infection alone or for treatment of TI patients suffering from HCV, HIV-1, and HBV. We also determined that *has-miR-30653p* fulfills the three important criteria with the three viruses. However, neither of the two miRNAs (i.e. *hsa-miR-3065-3p* and *hsa-miR-122)* exhibited 100% identity with HCV. *Hsa-miR-122* shows an imperfect identity at seed residue position #4 and *hsa-miR-3065-3p* shows an imperfect identity at seed residue position #7 in the seed sequence (2–8 bps) to the HCV genome (Table 1). The most important sequences that should be identical are the first four bps of the seed sequence (18) between the hsa-miRNA and the target sequence. Therefore, as shown in Table 1, in the case of *hsa-miR*-*3065-3p*, perfect homology is seen at the first four bps of the seed sequences (i.e. 2–5 bp), making it a relatively more important potential candidate for gene-based therapy against HCV alone and TI. However, *hsa-miR-122* exhibited imperfect homologies in the seed sequences for all three viruses, even for HCV (Table 1). Interestingly, both miRNAs target the GP1 site, which is an important step in viral entry (21, 24).

## Conclusion

We conclude that *hsa-miR-3065-3p* could be utilized as a potential target for antiviral therapeutics for TI patients and also in HCV mono-infected patients. Furthermore, miR-548 may be an alternate therapeutics agent in TI and HIV-1 HBV dual infected patients.
